# Differential Combination of Cytokine and Interferon- γ +874 T/A Polymorphisms Determines Disease Severity in Pulmonary Tuberculosis

**DOI:** 10.1371/journal.pone.0027848

**Published:** 2011-11-29

**Authors:** Ambreen Ansari, Zahra Hasan, Ghaffar Dawood, Rabia Hussain

**Affiliations:** 1 Department of Pathology and Microbiology, Aga Khan University, Karachi, Pakistan; 2 Masoomeen Trust Hospital, Karachi, Pakistan; Charité, Campus Benjamin Franklin, Germany

## Abstract

**Background:**

*Mycobacterium tuberculosis* infects nearly 1/3 of the world population and this reservoir forms the largest pool from which new cases arise. Among the cytokines, IFN-γ is a key determinant in protection against tuberculosis. Single nucleotide polymorphisms (SNPs) in *IFN-γ* gene (+874 T/A) which determine TT high (^hi^), AA low (^lo^) and TA intermediate (^int^) responder phenotypes have shown variable associations with tuberculosis disease outcome in different ethnic populations. The objective of the current study was to analyze *IFN*-γ gene combinations with other *IFN*-γ regulating cytokine genes (*IL-10*, *TNF –α*, *IL-6*) to see the effect of gene- combinations on disease severity outcome in pulmonary tuberculosis.

**Methods and Findings:**

Study groups comprised of pulmonary TB patients stratified according to lung tissue involvement into mild (Pmd = 74) or advance (Pad = 23) lung disease and compared with healthy controls (TBNA = 166). Genotype analysis was carried out using amplification refractory mutation system-PCR (ARMS-PCR). *IFN-γ* gene (+874 T/A) functional SNP combinations in *TNFα* (−308 G/A), *IL-10* (−1082 A/G) and *IL-6* (−174 G/C) were analyzed. Single gene analysis (Pearson χ^2^) showed a dominant association of IFN-γ TT ^hi^ genotype (p = 0.001) and T allele (p = 0.001) with mild disease. *IFN-γ* ^lo^ -*IL-10* ^lo^ genotype combination was associated with advanced disease (p = 0.002). *IFN-γ* ^hi^ –*IL-6* ^hi^ combination was associated with mild disease (p = 0.0005) while *IFN-γ* ^lo^ –*IL-6* ^int^ was associated with protection against both forms of pulmonary disease (p = 0.002).

**Conclusion:**

Our results show that a limited number of *IFN-γ* gene combinations with other cytokine functional SNPs determine the outcome of disease severity in tuberculosis.

## Introduction

Despite dramatic declines in industrialized countries, there are over 8 million new cases of *M. tuberculosis* (MTB) every year, 1.5 million deaths, and 40 million new infections [1]. Among the 20 high TB burden countries, Pakistan ranks 8^th^ despite >95% coverage with BCG vaccination at birth [2]. Of those exposed to tuberculosis, only about 30% are thought to develop the state of latent infection, during which the host remains clinically well, but bacilli survive within granulomae in a dormant state. Therefore, the normal human immune system is substantially equipped to control a TB infection [3]. Of those harboring a latent infection, on average, only 5–10% will progress to active clinical disease, approximately ½ within the first 2–3 years following infection [4]. There is a large body of evidence that genetic factors may play an important role in determining susceptibility as well extent of disease severity in TB.

Cytokines play an important role in orchestrating the immune response which is activated as a network of pro-inflammatory and down-regulatory cytokines derived from both T cells and macrophages and determine the disease outcome in TB. Interferon (IFN)-γ derived from activated Th1 cells plays a pivotal role in mycobacterial immunity [5]–[6]. However, *IFN-γ* gene (+874 T/A) single nucleotide polymorphisms (SNPs) which is related to the high (^hi^), intermediate (^int^) and low (^lo^) responder phenotype has shown variable effects in different populations [7]. Cytokines that affect the functionality of IFN-γ render the host susceptible to severe disseminated disease [8]–[12]. Among the pro inflammatory cytokines, Tumor Necrosis Factor-Alpha (TNF-α) in conjunction with IFN- γ plays a key role in the initiation, regulation, and maintenance of the inflammatory response generated by MTB. In autoimmune conditions and HIV patients, treatment with drugs aimed at blocking TNF-α have shown activation of latent TB [13]. *TNF*-α −308 G/A SNP, a functional polymorphism, has shown association with severe forms of malaria [14], leishmaniasis [15], and leprosy [16], in conjunction with raised levels of TNF-α in peripheral blood, but this SNP has shown no association with either disease susceptibility [17]–[18], [19], or severity in TB [20]. The effects of pro-inflammatory cytokines are counterbalanced by down-regulatory cytokines such as interleukin-10 (IL-10) which is produced by the activated macrophages, monocytes, Th2 [21], and T regs [22], in response to infection. The *IL-10* −1082 G SNP correlates with high responder phenotype after *in vitro* stimulation of T cells [23]. High levels of IL-10 may prevent collateral tissue damage particularly in the lung [22] during the chronic phase of pulmonary TB but may lead to disease activation during the early or latent phase of infection by down regulating both TNF-α and IFN-γ [24]. Data on association of *−1082* G/A SNP and TB is also highly variable [25].

A biallelic polymorphism has also been identified at position −174 (G/C) in the promoter region with the allele G found to be associated with higher IL-6 production [26]. To date no association has so far been reported with *IL-6* −174 SNP and TB [20], [27], although IL-6 is recently being recognized as part of the Th2 loop [28], which down regulates Th1 responses. We have also recently reported that endogenously activated high levels of IL-6 in conjunction with high levels of IL-4 was associated with disease progression in recently exposed household contacts of TB patients [29].

Because of the complexity of immune interactions which determine TB disease susceptibility and severity, multi-gene combination may give more meaningful insights than single SNP analysis in determining disease outcome [30]–[31]. Since IFN-γ is a key player in immunity to mycobacterial diseases we have investigated the combination of SNP +874 (T/A) with functional SNPs in other cytokines genes (*IL-10*, *TNF-α* and *IL-6*) to see if some of the variability in single gene association studies in different ethnic populations could be explained by multi-gene combination. Our results show that a few selected combinations of *IFN*-γ cytokine gene with other cytokine genes SNPs determine the outcome of disease severity in TB.

## Materials and Methods

### Study Groups

The patient population was drawn from a hyper-endemic area in Karachi (Kharadar). Healthy controls consisted of TB not affected (TBNA = 166) donors, drawn from within the community (HC = 78) and outside the community (EC = 88) and who had no co-morbids conditions, signs, symptoms or history of previous TB.

#### Inclusion criteria

The TB patient group comprised of pulmonary TB (PTB = 102) with involvement of lung parenchyma only. Exclusion criteria: Patients with immunosuppressive condition such as HIV, diabetes or on steroids for inflammatory conditions were excluded from the study. Diagnosis of PTB was confirmed by microscopy for presence of acid fast bacilli or sputum culture positivity (N = 55), radiology (N = 42) or clinical response to treatment (N = 5). PTB patients where radiological lung tissue involvement (N = 97) was available were further stratified into minimal (n = 10)/moderate (N = 64) or advanced (P ad = 23) disease according to non-HIV related TB guidelines for disease classification [32]–[33]. Five TB patients had been treated for PTB but radiological classification for disease severity was not available and therefore, excluded from the disease severity studies.

*IFN-γ* -874 SNP showed similar frequencies in PTB patients with minimal and moderate lung involvement and were grouped as mild disease (P md = 74) for analysis of cytokine gene polymorphisms. This study was approved by The Aga Khan University Ethical Review Committee (1452-PATH-ERC). Written consent was obtained from each participant.

### Tuberculin skin tests

Tuberculin skin test (TST) positivity was assessed by administering 5 tuberculin units intra-cutaneously on the volar surface of the right arm [34]. An induration of ≥10 mm after 48 hours was considered positive (TST+).

### Molecular Template Preparation

Two to five ml of blood was collected from all donors in ACD tubes (VWR Scientific, West Chester, PA, USA) and kept frozen at −35°C. Human genomic DNA was extracted from frozen whole blood at room temperature (25°C) after thawing and proper mixing of the tubes using Promega Wizard Genomic DNA Purification Kit (Promega Corporation Madison, WI, USA) according to the manufacturer's instructions. Extracted DNA was run on 0.8% agarose gel to check the quality of the DNA. Spectrophotometery was also done to check the concentration and purity of the DNA and stored at 4°C until further use.

### Molecular Methods

Primers were purchased from MWG-Biotech AG (Ebersberg, Germany). The sequences are given in Table S1. *IFN-γ* (rs2430561), *IL-10* (rs1800896) and *TNF-*α (rs1800629) genotyping were carried out using amplification refractory mutation system-PCR (ARMS-PCR) with human growth hormone (*HuGH*) or *β- actin* primers as internal controls to check the accuracy of PCR reactions. *IL-6* (rs1800795) genotyping was carried out using tetra-ARMS PCR. Amplified products (5 µl undiluted) were monitored by electrophoresis on agarose gel prepared in Tris-acetate EDTA (TAE) buffer containing 10 mg/ml ethidium bromide. Product bands were visualized on a UV-transilluminator and pictures were taken for the interpretation of genotypes.

### Confirmatory Sequencing Methodology

Sequencing methodology was used on a subset of samples (10–15% for each SNP) to confirm genotypes identified by ARMS and tetra-ARMS PCRs. The primers for sequencing reactions of *IFN*-γ and *IL-10* were designed using software Lasergene version 7.0 (DNAstar, Madison, WI, USA) while primers for *TNF-*α and *IL-6* were designed using web based software “BatchPrimer3” (Table S1). Amplified PCR products (25–30 ul) were sent to Macrogen (Macrogen Inc, Seoul, Korea) for sequencing. Sequencing results were analyzed by pair-wise alignments of the sequences using software “ClustalW version 1.83”. The allelic specificities of the cytokine gene SNPs were determined by a comparison of PCR results with nucleotide sequencing results of the alleles. Greater than 95% concordance was observed in all cases.

### Statistical Analysis

Epi- Info (version 6) was used to estimate the sample size with 95% confidence interval and 80% power of the study. We have used genotype frequencies in the Iranian population [35], for sample size calculations and added ∼20–25% for variability in frequency. Statistical Package for Social Sciences (SPSS version 19.0 IBM Corporation, NY, USA) was used. Groups were compared for differences in allele and genotypes frequencies by Pearson Chi squared or Fisher's exact tests, when the criteria for the chi-squared analysis were not fulfilled. P values of ≤0.05 were considered significant for both Pearson and Fisher's exact tests. For significant p-values, odds ratios (OR) with respective confidence intervals (95% CI) were also calculated. Odds Ratio (OR) was not available for all values because at least for one case, the value of the weight variable was zero. Linear-by-linear test were used to determine the corrected p-values for comparisons between groups and Bonferroni correction was applied when multiple comparisons were carried out. Multiple logistic regression analysis was applied to determine the effect of age and sex with genotypes. Hardy-Weinberg Equilibrium (HWE) was determined by applying the equation (p2+2pq+q2). *IFN*-γ +874 T/A genotype combinations with *IL-10* −1082 A/G, *TNF*-α -308G/A and *IL-6* −174 G/C were used to determine two gene combination effect. Nine possible genotype combinations were derived with *IFN-γ* genotypes for each of the cytokine genotypes. These combinations were then compared between controls and PTB patients using Pearson χ^2^ or Fisher's exact test.

## Results

The patients population was drawn from a hyperendemic area in Karachi (Kharadar) described in detail previously [34]. The age and gender distribution of donor groups included in the study are given in Table 1. There was no significant difference in these parameters between control and patient groups (χ square test). The rate of TST+ (> = 10 mm indurations) was 83% in PTB patients and 61% in healthy donors (TBNA). The high rate of TST+ in healthy control is not surprising as the setting of this study is in a high transmission pocket in Karachi [36]. Single nucleotide polymorphisms (SNPs) in *IFN-γ* gene (+874 T/A) which determine TT high (^hi^), AA low (^lo^) and TA intermediate (^int^) responder phenotypes were analyzed in relation to disease susceptibility and severity in pulmonary tuberculosis.

**Table 1:** Demographic Characteristics of TB affected and TB not affected donors.

Group	N	Age (mean)	SD ±1	Gender M∶F (ratio)	% TST+
TBNA	166	28.3	12.1	83/83 (1)	61
PTB	102	31.8	16.5	44/58 (0.76)	83
*a) Pmd*	74	32.4	15.9	33/41 (0.8)	
*b) Pad*	23	27	17	9/14 (0.64)	

Note: TBNA = TB not affected; PTB = Pulmonary tuberculosis; Pmd = Pulmonary patients with min/mod lung involvement; Pad = Pulmonary patients with extensive lung involvement.

TST+ = Tuberculin Skin Test≥10 mm induration.

### Cytokine SNP Genotypes and Allele frequencies in pulmonary tuberculosis patients

ARMS-PCR is a simple and reliable method for SNPs analyses as this methodology does not require restriction enzyme digestion or allele specific oligonucleotides and thus reduces sample handling. A concordance of >95% was observed between ARMS-PCR and sequencing (N = 40) (data not shown). To control for the population differences we tested healthy controls from within the community (HC = 78) and outside the community (EC = 88) and saw no differences in frequencies (IFN-γ, p = 0.590; IL-10, p = 0.296; TNF-α, p = 0.535; IL-6, p = 0.662). We therefore pooled the two control groups (TBNA = 166) for comparison with the tuberculosis patients. All genotypes were in Hardy-Weinburg -Equilibrium. Table 2 shows the distribution of genotype and allele for all four cytokines. We used additive and multiplicative models for analyses of association of genotypes and alleles with pulmonary TB and disease severity in pulmonary TB (Table 2).

**Table 2:** Cytokine gene polymorphisms in Pakistani Pulmonary Tuberculosis Patients.

Cytokines	SNP	TBNA (166)		PTB (102)		Pmd (74)		Pad(23)	
		n	%	n	%	n	%	N	%
IFN-γ GN	TT	21	12.65	24	23.53	22	29.73	2	8.7
(+874 T/A)	TA	80	48.19	48	47.06	34	45.95	11	47.83
	AA	65	39.16	30	29.41	18	24.32	10	43.48
χ^2^					5.49		10.44		0.31
p =					**0.019**		**0.001**		0.577
IFNγ Allele	T	122	36.75	96	47.06	78	52.7	15	32.61
(+874 T/A)	A	210	63.25	108	52.94	70	47.3	31	67.39
χ^2^					5.56		10.7		0.3
p =					**0.018**		**0.001**		0.584
OR					1.53		1.92		0.83
95% CI					1.07–2.18		1.30–2.84		0.43–1.60
IL-10 GN	AA	31	18.67	23	22.55	17	22.97	6	26.09
(−1082 A/G)	AG	118	71.08	64	62.75	48	64.86	11	47.83
	GG	17	10.24	15	14.71	9	12.16	6	26.09
χ^2^					0.01		0.1		0.46
p =					0.933		0.757		0.498
IL10 Allele	A	152	45.78	94	46.08	66	44.59	23	50
(−1082 A/G)	G	180	54.22	110	53.92	82	55.41	23	50
χ^2^					0		0.06		0.29
p =					0.947		0.809		0.591
OR					1		1.05		0.85
95% CI					0.69–1.4		0.71–1.55		0.46–1.57
TNFα GN	GG	83	50	51	50	41	55.41	8	34.78
(−308 G/A)	GA	83	50	51	50	33	44.59	15	65.22
	AA	0	0	0	0	0	0	0	0
χ^2^					0		0.6		1.86
p =					1		0.44		0.172
TNFα allele	G	249	75	153	75	115	77.7	31	67.39
(−308 G/A)	A	83	25	51	25	33	22.3	15	32.61
χ^2^					0		0.41		1.22
p =					1		0.523		0.27
OR					1		0.86		1.45
95% CI					0.70–1.50		0.54–1.36		0.75–2.82
IL-6 GN	GG	100	60.24	74	72.55	51	68.92	18	78.26
(−174 G/C)	GC	56	33.73	24	23.53	19	25.68	5	21.74
	CC	10	6.02	4	3.92	4	5.41	0	0
χ^2^					3.79		1.22		3.31
p =					(0.052)		0.27		0.069
IL-6 allele	G	256	77.11	172	84.31	121	81.76	41	89.13
	C	76	22.89	32	15.69	27	18.24	5	10.87
χ^2^					*4.07*		1.31		3.47
p =					*0.044*		0.252		0.063
OR					*0.63*		0.75		0.41
95% CI					*0.40–0.99*		0.46–1.23		0.16–1.08

Note: Abbreviations for groups are as described in table 1. Group N is given in brackets. Groups were compared with TBNA. Pearson χ2 or Fisher exact test was performed to determine group differences. Linear by linear corrected p values are shown. Significant differences (p<0.05) are in bold. Association with protection is given in italics.

### *IFN-γ* (+874 T/A) SNP shows significant association with pulmonary disease

A significant association of TT^hi^ genotype (p = 0.019; TST+ corrected p = 0.024) as well as the T allele (p = 0.018, TST+ corrected p = 0.023) was observed with PTB. This association was restricted to P md (TT genotype p = 0.001, TST corrected p = 0.002; and T allele, p = 0.002) suggesting that the presence of this genotype increases the risk of P md (OR 1.92; CI = 1.07–2.84) (Table 2). As previously reported [37], no association was found with Pad (p = 0.591). T allele therefore shows association with the less severe form of pulmonary TB (Table 2).

### *IL-10* (−1082 A/G), *TNF-α* (−308 G/A) SNPs

*IL-10* (−1082A/G) and *TNF-*α (−308G/A) genotype and allele frequencies showed no significant differences between disease and control subjects in relation to either site (PTB) or severity of pulmonary disease (Table 2). Correction for TST+ controls gave similar results (Table 2).

### *IL-6* (−174G/C)

The genotype frequency of *IL-6* (−174G/C) indicated a trend (p = 0.052) with lower frequency of CC in the PTB group but was not significant when corrected for TST+ (p = 0.179). This trend achieved statistical significance (p = 0.044) when alleles were compared, with the C allele showing lower frequency in PTB patients and decreased the risk of PTB disease (OR = 0.63; CI = 0.40–0.99) compared to healthy controls. However this trend again was not significant when corrected for TST+ (p = 0.147).

In addition to the multiplicative and additive models, we also analyzed the dose effect of genotypes using the dominant and recessive model for each of the 4 genotypes (Table 3). The effect of *IFN*- γ +874 T allele was a dominant trait while the *IL-10* A allele showed a recessive trait. *TNF*-α and *IL-6* were not significant in this model (Table 3).

**Table 3:** Effect of cytokine gene dose on severity of pulmonary tuberculosis.

Cytokines	SNP Position	Genotypes	PTB (102)	PMN+PMD (74)	PAD (23)
			P-values	P-values	P-values
IFNγ	(+874) T→A	Additive	**0.046**	**0.003**	
		Dominant		**0.026**	
		Recessive	**0.021**	**0.001**	
		Multiplicative	**0.018**	**0.001**	
IL-10	(−1082) G→A	Additive			**0.041**
		Dominant			**0.03**
		Recessive			
		Multiplicative			
TNFα	(−308) A→G	Additive			
		Dominant			
		Recessive			
		Multiplicative			
IL-6	(−174) G→C	Additive			
		Dominant			
		Recessive	**0.041**		
		Multiplicative	**0.043**		

Note: Abbreviations for groups are as described in table 1. Group N is given in brackets.

Groups were compared with TBNA. Pearson χ2 or Fisher exact test was performed to determine group differences. Linear by linear corrected p values are shown.

### *IFN*- γ +874 T/A gene combination with *IL-10*, *TNF*-α, *IL-6* on PTB disease severity

Deficiencies in IFN- γ and cytokines regulating or modulating the IFN- γ response have been shown to have profound effect on mycobacterial immunity and in some cases has resulted in Mendelian Susceptibility to Mycobacterial Diseases (MSMD) [38]. We therefore analyzed the effect of *IFN- γ* +874 SNPs in combination with other promoter region SNPs in cytokines *IL-10*, *TNF*-α, *IL-6* (Table 4). For each cytokine, nine combinations were generated with *IFN- γ* +874 T/A SNPs. The frequency (%) and number (n) is given for all groups but statistics for only significant values are shown (Table 4). Uncorrected p values<0.005 were considered significant as there were 9 possible combinations (Bonferroni correction).

**Table 4:** Tables 4. IFN -γ Genotype combination: frequency and association with tuberculosis disease susceptibility and severity.

IFNγ-IL10	TBNA % (n = 166)	PTB% (n = 102)	p-value; χ2	OR (95% CI)	Pmd % (n = 74 )	P-value; χ2	OR (95% CI)	P ad % (n = 23)	*P-value; χ2	OR (95% CI)
TT^High^-AA^High^	6.42	8.82			4.48			4.35		
**TT^High^-AG^Int^**	8.56	10.78			5.97			0.00	**0.002; 29.42**	
TT^High^-GG^Low^	3.21	5.92			2.99			4.35		
TA^Int^-AA^High^	5.88	7.84			2.99			8.7		
TA^Int^-GA^Int^	38.5	34.31			43.28			30.43		
TA^Int^-GG^Low^	3.74	4.9			2.99			8.7		
AA^Low^-AA^High^	5.88	5.88			5.97			13.04		
AA^Low^-GA^Int^	24.06	17.65			29.85			17.39		
**AA^Low^-GG^Low^**	3.74	5.88			1.49			13.04	**0.0022; 5.21**	**3.59 (1.12–11.41)**
**IFNγ-TNF**α										
TT^High^-AA^High^	0.00	0.00			0.00			0.00		
**TT^High^-GA^Int^**	5.42	12.75	0.048;3.92	2.84 (0.97–8.29)	14.86	**0.018; 5.56**	**3.35 (1.17–9.62)**	8.7		
TT^High^-GG^Low^	7.23	10.78			14.86			0	**0.01; 27.25**	
TA^Int^-AA^High^	0.00	0.00			0.00			0.00		
TA^Int^-GA^Int^	26.51	24.51			21.62			30.43		
TA^Int^-GG^Low^	21.69	22.5			24.32			17.39		
AA^Low^-AA^High^	0.00	0.00			0.00			0.00		
*AA^Low^-GA^Int^*	18.07	12.75			8.11	*0.036; 4.42*	*0.4 (0.16–0.96)*	26.09		
AA^Low^-GG^Low^	21.08	*16.67*			*16.22*			*17.39*		
**IFNγ-IL6**										
**TT^high^-GG^high^**	4.22	15.69	0.005;8.00	4.57 (1.47–14.21)	20.27	**0.0005; 12.12**	**6.0 (1.97–18.27)**	4.35		
TT^high^-GC^Int^	7.83	6.86			8.11			4.35		
TT^high^-CC^low^	0.60	0.98			1.35			0.00		
TA^Int^-GG^High^	32.53	30.39			27.03			34.78		
TA^Int^-GC^Int^	11.45	14.71			16.22			13.04		
TA^Int^-CC^low^	4.22	1.96			2.70			0.00		
**AA^low^-GG^High^**	23.49	26.47			21.62			39.13	0.014; 25.98	2.14 (1.16–3.96)
***AA^Low^-GC^Int^***	14.46	*1.96*	*0.002;9.78*	*0.13 (0.03–0.57)*	*1.35*	***0.0005; 12.18***	***0.06 (0.01–0.48)***	4.35	***0.013; 6.1***	***0.26 (0.08–0.81)***
AA^Low^-CC^Low^	1.2	0.98			1.35			0.00		

Note: Abbreviations same as in table 1. Number in columns indicates the frequency as percent of total in each group given in brackets.

*Uncorrected p values.

P<0.005 (underlined) was considered significant after Bonferroni correction for multiple comparisons. Values in italics indicate protection and in bold indicate susceptibility.

1. **Combinations of IFN- γ +874 T/A with IL-10 -1082 A/G SNPs.** Although *IL-10* -1082 A/G SNP showed no association with pulmonary TB (Table 2) when analyzed singly but had an effect in combination with *IFN- γ* +874 T/A SNP (Table 4). Absence of *IFN*- γ +874 ^hi^ *IL-10* ^int^, resulted in increased risk of Pad (p = 0.002). In addition presence of *IFN- γ* +874 ^lo^ *IL-10* ^lo^ also increased the risk of Pad (p = 0.0022, OR = 3.59, CI = 1.12–11.41) (Table 4). These results were statistically significant even when strict bonferroni correction was applied. IFN-γ is a potent pro-inflammatory cytokine. Therefore, high IFN-γ levels have to be counterbalanced with down regulatory *IL-10* to reduce collateral damage. These results are therefore in line with the biological functions of IFN-γ and IL-10. No association was observed with P md for any of the *IFN*- γ-*IL-10* genotype combinations (Table 4).
2. **Combinations of IFN- γ +874-T/A with TNFa −308 G/A SNPs.** TNF-α, a pro inflammatory cytokine in conjunction with IFN- γ has been shown to play an important role in granuloma formation and disease localization [39]. When gene combinations for these two cytokines were analyzed (Table 4), it was interesting to note that weak associations were noted for *IFN*- γ ^hi^ – *TNF-*α ^int^ which restricted the disease to Pmd (p = 0.018, OR = 3.35, CI 1.17–9.62) while a decreased frequency of *IFN*- γ^lo^ – *TNF-*α ^int^ was weakly associated with protection in Pmd (p = 0.036, OR = 0.4, CI = 0.16–0.96) (Table 4) suggesting that *IFN*- γ ^hi^ may be responsible for tissue damage in Pmd. This is further supported by the observation that lack of *IFN- γ* ^hi^ – *TNF-*α ^lo^ increases the risk of Pad (p = 0.01) which is again in line with the role of these two cytokines. Although these associations are weak and become insignificant when strict bonferroni correction was applied, these results to some extent may explain the controversial association of *IFN- γ* ^lo^ and *IFN- γ* ^hi^ alleles with either susceptibility or protection for PTB in different ethnic population which may be related to the differential frequencies of *IFN-γ* SNPs [7], in relation to other cytokine gene SNPs.
3. **Combination of IFN- γ +874-T/A with IL-6 −174 G/C SNPs.** IL-6 is part of the acute phase responses but its biological functions are just being defined in the activation of the Th2 network [28]. Single polymorphisms with *IL-6* −174 SNPs in different studies have shown no association with PTB [27], [20]. It was therefore interesting to note clear cut associations with disease protection and susceptibility when the combinations of this cytokine with *IFN- γ* were analyzed. There was one combination, *IFN-γ* ^lo^ -*IL-6* ^int^ which was decreased across the pulmonary disease spectrum and was associated with decreased risk of pulmonary TB (Table 4). This combination (*IFN*-*γ* ^lo^ *IL-6* ^int^) also showed greater protection with moderate disease (Pmd ; p = 0.005; OR = 0.06) compared to advanced disease (Pad; p = 0.013, OR = 0.26). Increased frequency in *IFN- γ* ^hi^ - *IL-6* ^hi^ was associated with increased risk of Pmd (p = 0.0005; OR = 6.0, CI = 1.97–18.27) but not Pad (Table 4). In summary there were only a limited set of combinations with *IFN-γ* which showed significant association with either susceptibility or severity of pulmonary disease. Interestingly different gene combinations were associated with the less severe (*IL-6*; Pmd) or more severe form (*IL-10*; Pad) of pulmonary disease.

## Discussion

Single nucleotide functional polymorphisms in cytokine genes have shown variable associations with TB site and severity of disease in different populations and even within the same study population [7]. Most studies have not differentiated TB disease by site or severity and therefore it is difficult to evaluate the differences in results. The strength of our study groups was that we have focused on a single site (lung) and also stratified the disease severity in this site. In addition we have analyzed gene combinations with *IFN-γ* gene which plays a key role in TB disease susceptibility and severity. Combinations of cytokines SNPs with *IFN-γ* +874 T/A SNP exert considerable influence on outcome of severity in TB. Our results may be reflecting polygenic aspects of predisposition to disease severity and active disease. [40], [31], [38].

The functional SNP at position +874 (T/A) is located at the 5′-end of a CA repeat at the first intron of human *IFN-γ* gene. The T allele correlates with high *IFN-γ* expression. Transcriptional factor, NF-κB binds preferably to DNA containing *IFN-γ* +874 T allele, and increases the expression *IFN-γ* gene. *IFN-γ* +874 single gene association studies have shown variable results in different ethnic populations with AA phenotype associated with pulmonary disease in several studies [41], [23], [12], [42]–[45]. Interestingly, in two studies T allele (High producer allele) was associated with either localized pleural TB disease [20], or with less severe form of the pulmonary TB but not with advanced pulmonary disease [37]. These differences in observations may be due to the heterogeneity in SNPs frequencies in different populations [35], and in particular differences in frequencies of *IFN-γ* +874 *TT* genotype which varies from nil to 40 percent (see Table S2).

When we carried out combination analysis in the stratified groups, only 1/27 possible combinations showed any significant association with protection across the pulmonary disease spectrum (Table 4). Furthermore, our results indicate that only a limited number of combinations show meaningful association with disease severity. The combination of *IL-6* ^hi^ with *IFN-γ* ^hi^ or *IFN-γ* ^lo^ was the most decisive in determining disease severity. This observation is in line with the recently described function of IL-6 as part of the Th2 loop [28]. The other interesting combination which showed clear cut differences in disease severity was *IFN-γ* ^hi^ or *IFN-γ* ^lo^ with *IL-10* SNPs. *IFN-γ* ^hi^ in the presence of *IL-10* ^int^ showed association with pulmonary advanced disease while *IFN-γ* ^lo^ with *IL-10* ^lo^ also showed an association with pulmonary advanced disease indicating the critical role of IL-10 in reducing collateral lung tissue damage. Combination of *IFN-γ* ^hi^ or *IFN-γ* ^lo^ with *TNF-α* ^hi^ or *TNF-α* ^lo^ SNPs showed only weak associations at best but it was clear that different combinations were associated with either less or more severe disease.

The immune response to *M. tuberculosis* infection is broad-ranging and complex. *M. tuberculosis* has evolved many strategies for circumventing the host's immune defenses. CD4-activated IFN-γ appears to be essential [5], [46], though not sufficient, for maintenance of latency [47]. TNF-α plays an important role in maintaining the integrity of granulomae, and preventing reactivation [13], and IL-10 appears to be important in reducing collateral damage and determination of severity [22]. In household contact biomarker studies, early progression to disease has been associated with alterations in the production of regulatory Th-2 cytokines, such as IL-4 [29], and IL-10 [48], Thus, the balance of pro inflammatory and suppressive immune responses appears to be important in controlling protection and or disease severity in tuberculosis. Our results with cytokine high and low producer phenotypes are consistent with the biological functions defined for these cytokines.

In conclusion our study highlights the importance of careful stratification of patient groups according to disease severity in association studies. Secondly, single gene association studies of even the most important player i.e. *IFN*-γ in mycobacterial immunity may lead to misleading results. Therefore, additional multi-loci gene interaction studies are warranted in different ethnic populations to understand the significance of different dominant or recessive phenotype effects in disease outcome in TB.

## Supporting Information

Table S1 **Primer Sequences, SNP positions and detection methods used for genotypes and alleles determination.**
(DOCX)

Table S2 **Genotype and allele Frequency of cytokine IFN-γ (+874 T/A) SNP in Different Population.**
(DOCX)
