# Supplementary material for: Differential Combination of Cytokine and Interferon- γ +874 T/A Polymorphisms Determines Disease Severity in Pulmonary Tuberculosis
Source: PLoS One. 2011 Nov 29;6(11):e27848. doi: 10.1371/journal.pone.0027848 (PMC3226558; doi:10.1371/journal.pone.0027848)
Supplement: Table S1 — Primer Sequences, SNP positions and detection methods used for genotypes and alleles determination. (DOCX) [file pone.0027848.s001.docx]

Table S1. Primer Sequences, SNP positions and detection methods used for genotypes and alleles determination (mat & material supp Table)
