# Supplementary material for: Differential Combination of Cytokine and Interferon- γ +874 T/A Polymorphisms Determines Disease Severity in Pulmonary Tuberculosis
Source: PLoS One. 2011 Nov 29;6(11):e27848. doi: 10.1371/journal.pone.0027848 (PMC3226558; doi:10.1371/journal.pone.0027848)
Supplement: Supplementary file 2 [file pone.0027848.s002.docx]

Table S2 Genotype and allele Frequency of cytokine IFN-γ (+874 T/A) SNP in Different Population

rs # rs2430561

Ancestral Allele 'T'

					Genotype %			Alleles %	
**Author**	**Year**	**Ethnicity**	**Donors**	**N=**	**TT**	**TA**	**AA**	**T**	**A**
Anuradha et al	2008	Indian	Controls(BCG-)	45	35.6	51.1	13.3	61.2	38.9
Larcombe et al	2008	Caucasians	Controls	91	31	44.0	25.0	53.0	47.0
Lio et al	2002	Sicilian	Controls	97	25.8	48.4	25.8	50.0	50.0
Moran et al	2007	Caucasians	Controls	64	25	48.0	27.0	51.8	48.2
Amim et al	2007	Brazilian	Controls	266	25	33.0	41.0	41.5	57.5
Sallakci et al	2007	Turkish	Controls	115	23	50.0	27.0	47.8	52.2
Poli et al	2002	Italian Caucasian	Controls	363	21.2	46.8	32.0	44.7	55.3
Etokebe et al	2005	Corotian	Controls	519	19.8	54.3	25.8	47.0	53.1
Lopez-Maderuelo et al	2003	Spanish	Controls	100	19	50.0	31.0	44.0	56.0
Oral et al	2006	Turkish	Controls	50	16	42.0	42.0	37.0	63.0
Vidyarani et al	2006	Indian	Controls	127	15.8	40.9	43.3	36.0	64.0
Ansari et al	2008	Pakistani	Controls	166	13.3	46.3	40.4	27.7	72.3
Selvaraj et al	2008	Indian	Controls	188	12.9	42.7	44.4	34.0	66.0
Tso et al	2005	Chinese Hong Kong	Controls	451	12.2	42.1	45.7	33.3	66.7
Rossouw et al	2003	South African colored	Controls	235	11	42.0	47.0	32.0	68.0
Larcombe et al	2008	Canadian (Cree)	Controls	42	11	30.0	59.0	26.0	74.0
Ding et al	2008	Chinese	Controls	310	10.3	36.8	52.9	28.7	71.3
Moran et al	2007	Hispanics	Controls	98	10	27.0	63.0	20.4	79.6
Wit et al	2011	South African	Controls	318	10	39.0	50.0	20.1	79.9
Moran et al	2007	African American	Controls	174	7	37.0	56.0	14.7	85.3
Anuradha et al	2008	Indian	Controls (BCG+)	45	6.7	42.2	51.1	27.8	72.2
Mosaad et al	2010	Eygyptian	Controls	118	5.1	50.8	44.1	10.4	89.6
Buijtels et al	2008	Zambian	Controls	119	2.6	19.1	78.3	12.2	87.8
Cooke et al	2006	Gambian	Controls	594	2.2	27.9	69.9	16.2	83.9
Fitness et al	2004	South Africans	Controls	703	2	31.0	66.0	17.5	82.5
Wang et al	2010	Chinese	Controls	527	1.9	17.3	80.8	3.8	96.2
